# Social Media Overload and Anxiety Among University Students During the COVID-19 Omicron Wave Lockdown: A Cross-Sectional Study in Shanghai, China, 2022

**DOI:** 10.3389/ijph.2022.1605363

**Published:** 2023-01-10

**Authors:** Yangyang Wang, Jian Xu, Tian Xie

**Affiliations:** ^1^ China Institute for Urban Governance, Shanghai Jiao Tong University, Shanghai, China; ^2^ School of International and Public Affairs, Shanghai Jiao Tong University, Shanghai, China; ^3^ School of Media and Communication, Shanghai Jiao Tong University, Shanghai, China

**Keywords:** anxiety, risk perception, information overload, social overload, information strain, health management during urban governance

## Abstract

**Objectives:** The increase in the intensity of social media use during the COVID-19 lockdown has affected mental health. Therefore, it is of practical implications to explore the association between social media overload and anxiety and the underlying mechanisms.

**Methods:** Using data from 644 university students during the COVID-19 blockade in Shanghai from March to April 2022, the chain mediation model of information strain and risk perception of omicron between social media overload and anxiety was then tested using the macro PROCESS4.0 tool.

**Results:** The findings showed that social media overload (including information overload and social overload) was positively associated with anxiety. This relationship was mediated by information strain and risk perception of Omicron. A chain mediating role of information strain and risk perception of Omicron has also been proved in this study.

**Conclusion:** Social media overload has a positive effect on anxiety by increasing information strain and risk perception of Omicron. This study provides some implications for future interventions on how to use social media properly for mental health during the pandemic and health management of urban governance.

## Introduction

The strain of Omicron has been spreading rapidly all over the world since its first detection in November 2021[1]. In April 2022, a severe Omicron outbreak broke out in Shanghai, infecting more than 600,000 people. Chinese government adopts a zero-Covid strategy and imposes strict lockdown measures. Under this policy, tens of thousands of university students have been banned from campus and dormitories in Shanghai for more than a month. University students were more vulnerable than adults [2], they reported a higher level of anxiety during the quarantine in many countries [3–5]. A longitudinal study of 35,516 Chinese university students found that the symptom of anxiety increased from 11.5% to 18.3% after 4-month home isolation [6].

The COVID-19 pandemic is not only a global health public event but also a kind of “infodemic” [7]. Social media plays an important role as the main source of information during the COVID-19 pandemic. However, the dark side of social media can’t be ignored. During the pandemic, compulsive social media use, social media addiction[8] and social media infodemic[9] have emerged as widespread issues. In addition, social media use may also be associated with negative emotional and psychological impacts such as worry [10], fear [11], stress [12], anxiety [13] and depression [14]. Another problem with social media use during the pandemic was social media overload. According to the bounded rationality theory, social media overload refers to an individual’s social media processing capacity falling short of the massive information and social input [15,16]. Users spend a lot of time on social media dealing with a constant stream of information from a variety of sources. Besides, individuals at the center of an outbreak provide too much social support to others on social media. Drawing on social support theory, Maier et al. [17] defined this phenomenon as social overload [17]. In general, social media is loaded with information and social demands during the outbreak, which results in information overload and social overload [18].

## Study Rationale and Hypothesis Development

Some scholars have explored the direct relationship between overload and mental health. One study revealed that three stressors of SoLoMo (social-local-mobile) services including information overload, social message overload, and perceived surveillance have significant and direct impacts on users’ anxiety[19]. Another study found that COVID-19 information overload was positively associated with anxiety and depression [20]. However, these studies were not targeted at social media, especially the further exploration of social overload. Some studies have explored how social media overload affects behavioral consequences through its impact on negative psychological factors [21,22]. However, the mechanism of social media overload on mental health is still lacking in in-depth research and discussion, and there is a lack of study in the context of public health events. To fill the gap above, the manuscript conducted an online survey to explore the mechanism of social media overload on college students’ anxiety.

### Social Media Overload and Anxiety

According to cognitive load theory, once the resources to be dealt with exceed the total amount of personal cognition and become overloaded, it will affect personal emotions. Prior research showed that social media overload may result in negative emotions. Information overload brought redundancy and poor quality of information, people have to spend time sifting through valid information and identifying the authenticity of the information, which may lead to negative emotions [23]. Some studies have identified relationships between information overload and negative psychological health outcomes, such as depression [24], Wellbeing [25], and anxiety [20]. Social media breaks down the boundaries of space and time, and the lockdown policy reduced face-to-face socializing, and increased online social connections [26]. It has been found that information overload and social message overload were positively related to anxiety, however, social support overload had no significant effect on anxiety [19]. Another study holds a different opinion that social and information overload had no direct relationship with psychological outcomes [23]. Therefore, the association between social media overload and anxiety is still to be confirmed. To sum up, we propose the following hypotheses:

H1a: Information overload has a significant positive effect on anxiety;

H1b: Social overload has a significant positive effect on anxiety.

### The Mediating Role of Information Strain

The concept of information strain comes from technostress, which refers to fatigue, invasion, and other psychological pressures brought about by the continuous flow of information [27]. It has been found that technology stress was positively related to negative emotions such as anxiety, and fatigue [28,29]. Bermes examined the mechanism of fake news sharing among social media users and indicated that information overload was positively related to information strain [30]. Generally, higher cyber-based information overload predicted a higher level of stress [31]. Social overload has been viewed as a stressor in different studies. One study pointed out that social overload induced emotional exhaustion [32]. College students spend much time and energy on social activities related to COVID-19, when there are too many social connections to keep up, information strain may increase. Even though no evidence has examined the association between information strain and anxiety, previous studies showed that job stress significantly affects anxiety disorder [33,34]. Accordingly, we propose the following hypotheses:

H2a: Information strain mediates the relationship between information overload and anxiety;

H2b: Information strain mediates the relationship between social overload and anxiety.

### The Mediating Role of Risk Perception of Omicron

The social amplification of risk framework states that the transmission of risk information is an important stage of society amplification risk, and social media act as a “social amplification station” in the process [35,36]. And the amplification of risk has an impact on individual psychological factors. Anxiety is a negative emotion about the future that will arise when people experience an impending risk [37]. Recent research from different countries has examined the association between COVID-19 risk perception and anxiety, such as Swiss [38], Nigeria [39], and China[40]. The massive flow of risk-related information spreads through the population and affects risk perception [35,41]. Elmer et.al. [42] pointed out that the stressors of university students on social media have shifted from fears of missing out on social life to being anxious about their families, friends, health, and future during the pandemic [42]. Another study revealed that getting information about the virus from friends and family was a significant predictor of risk perception [43]. In general, social interaction affects people’s risk perception [44]. These studies proved that both information and social overload are associated with risk perception, and risk perception may affect anxiety. Therefore, we propose the following hypothesis:

H3a: Risk perception of omicron mediates the relationship between information overload and anxiety;

H3b: Risk perception of omicron mediates the relationship between social overload and anxiety.

### The Chain Mediating Role of Information Strain and Risk Perception of Omicron

Based on the risk-as-feelings hypothesis, emotions play a prominent role in risk perception [45]. The “experiential system” regards risk as a kind of feeling which relies on images [46]. However, prior empirical evidence on the association between information strain and risk perception is scarce. It has been found that higher levels of perceived risk were influenced by psychological stress [47]. Yang and Lin [19] revealed that technostress may result in symptoms of anxiety and tension [19]. According to a study on the perception of terrorist attack risk, the relationship between exposure to terrorist images and psychophysiological stress response has an impact on perceptions of the possibility of future attacks [48]. This research is similar to our research ideas. When the information overload and social overload on social media about the pandemic cause stressful reactions in users, the related risk perception will be significantly improved. Therefore, we infer that information strain positively affects perceived risk, and the following hypothesis has been proposed:

H4a: Information overload positively influences anxiety through the chain mediating role of information strain and risk perception of Omicron;

H4b: Social Overload positively influences anxiety through the chain mediating role of information strain and risk perception of Omicron.

**FIGURE 1:**
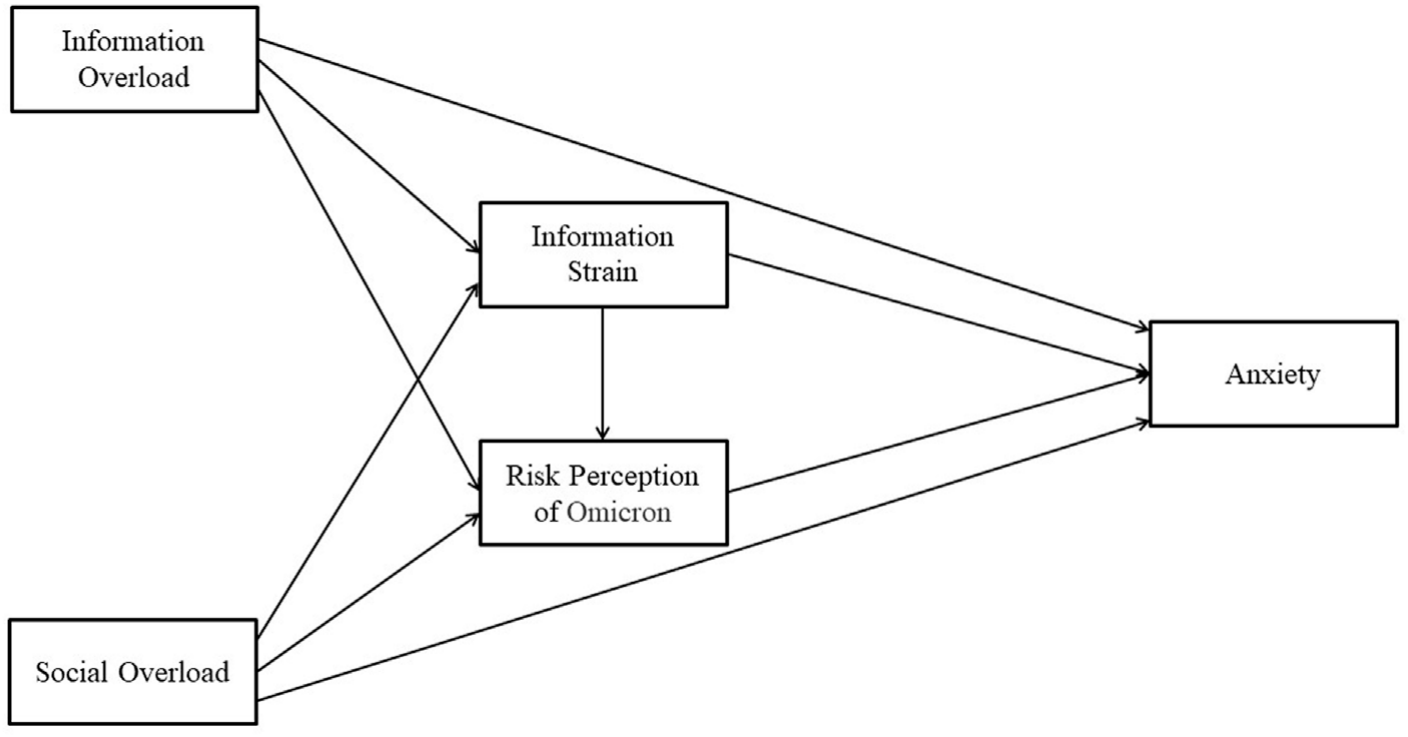
Theoretical model of the relationship between social media overload and anxiety (Shanghai, China. 2022).

The theoretical model of the relationship between social media overload and anxiety was constructed from the hypotheses proposed above, as shown in Figure 1.

## Methods

### Study Design, Population, and Sampling

From 25 March 25–13 April 2022, we conducted a Shanghai city-wide online anonymous survey using Wen Juan Xing. Wen Juan Xing which is equivalent to Qualtrics, SurveyMonkey, or CloudResearch, provides online questionnaire design and survey services for companies, research institutions, and individuals. It has been used in some academic studies [49–53]. Our survey was conducted during the Omicron wave lockdown in Shanghai in 2022. A random sampling procedure stratified by age, gender, and education was used to match a database sample of university students in Shanghai, followed by an anonymous self-reported online survey in Chinese. A random sample of 800 respondents was selected and 644 valid participants were retained after excluding incomplete and invalid questionnaires. Our study was approved by the Ethics Review Committee for Scientific and Technological Research Involving Human Beings of Shanghai Jiao Tong University (H2022200I).

### Measures

The main variables were measured from the scales that were being used. In the process of designing our questionnaire, the translation of the scale questions, wording and semantics were adapted to use the expression more in line with the conventions of the Chinese context. In addition, all variables were measured using a five-point Likert scale (1 = strongly disagree to 5 = strongly agree).

#### Anxiety

Anxiety was measured using the GAD-7 item scale by Spitzer et al [54]. The scale contains seven items and was measured using a five-degree Likert scale. Its Cronbach’s α is .811, and the results of confirmatory factor analysis (CFA) showed that the factor loading values for the seven items were .803, .829, .681, .533, .589, .628, and .438, respectively.

#### Social Media Overload

The measurement of social media overload is referred to Fu et al.'s study [55], and is divided into two dimensions: information overload and social overload. Information overload is measured using the scale developed by Zhang et al [56], which contains four measurement items. Its Cronbach’s α is .832, and the factor loading values from CFA for the four items were .702, .776, .797, and .695, respectively. Social overload was measured using the scale developed by Maier et al [17], which contains four items. Its Cronbach’s α is .772, and the factor loading values from CFA for the four items were .653, .770, .610, and .756, respectively.

#### Information Strain

Information strain was measured using the scale developed by Bermes et al ([57], p. 19), which contains four measure items. Its Cronbach’s α is .747, and the factor loading values from CFA for the four items were .555, .586, .762, and .761, respectively.

#### Risk Perception of Omicron

Risk perception of Omicron was measured using the scale developed by Zhuang et al [58]. The measurement of risk perception is based on the perspective of consequence, mainly from four aspects: society, individual health, family work, and family health. There are four items in total, and Cronbach’s α is .795. In addition, the factor loading values from CFA for the four items were .578, .506, .850, and .836, respectively.

Furthermore, confirmatory factor analysis (CFA) was performed for each construct and the corresponding factor loading values were reported above, and the results demonstrated that the validity of the study variables was satisfied for further analysis. Specifically, the fitted χ^2^/df value of the modified CFA model was 2.155, and the NFI, CFI, TLI, GFI, and IFI values were .927, .959, .952, .939, and .959, respectively, with model metrics greater than .9, which met the criteria.

### Control Variables

Based on the characteristics of the respondents and the context of the study, socio-demographic, school characteristics, life status, and social media use variables were selected as control variables in this study. Socio-demographic characteristics included gender (Female, Male), age, and university stage (Bachelor’s degree in progress, Master’s degree in progress, Doctorate in progress). School characteristics included school outbreaks (Yes, No), university type (General University; Key University; Top University), and on-campus accommodation (Yes, No). Life status included physical status (COVID-19 negative; COVID-19 positive) and residence status (Living alone; Others). Social media use included social media usage (Never use; 1 h and below; 1∼3 h; 4∼5 h; 5 h and above).

### Statistical Analysis

Descriptive analysis and Common Method Bias were done for all variables, and correlation analysis was conducted for the main variables. The mediating effects of information strain and risk perception of Omicron (H1a, H1b; H2a, H2b; H3a, H3b; H4a, H4b) were tested using model 6 of the macro PROCESS4.0 tool [59]. All the above analyses were performed with the help of SPSS26.

## Results

### Descriptive Analysis of the Overall Sample

Table 1 shows that the respondents of this survey were 383 female college students and 261 male college students, accounting for 59.5% and 40.5%, respectively. Most respondents were under 23 or younger, 80.4% were undergraduate students, 63.2% of respondents encountered COVID-19 happened on their campus, 61% (393) were at top universities, and 64.3% lived on campus dormitories. Only 7% of respondents had been infected with the COVID-19 Omicron virus, and 11.8% were living alone. A detailed description of the full sample characteristics is given in Table 1.

**TABLE 1:** Descriptive statistics of all variables (N = 644) (Shanghai, China. 2022).

Variables	Type	Number of people	Proportion (%)
Gender	Female	383	59.5
	Male	261	40.5
Age	<20	118	18.3
	20∼21	196	30.4
	22∼23	194	30.1
	24∼25	70	10.9
	26∼30	64	9.9
	30∼32	2	0.3
University stage	Bachelor’s degree in progress	518	80.4
	Master’s degree in progress	96	14.9
	Doctorate in progress	30	4.6
School outbreaks	No	237	36.8
	Yes	407	63.2
University type	General University	186	28.9
	Key University	65	10.1
	Top University	393	61.0
On-campus accommodation	Yes	414	64.3
	No	230	35.7
Physical status	COVID-19 negative	637	98.9
	COVID-19 positive	7	1.1
Residence status	Living alone	76	11.8
	Others	568	88.2
Social media usage	Never use	7	1.1
	<1 h	80	12.4
	1∼3 h	292	45.3
	4∼5 h	183	28.4
	5 h <	82	12.7

### Common Method Bias

The data were tested for possible Common Method Bias using the method suggested by Podsakoff et al. [60]. The variables were tested together using SPSS26 for Harman’s single-factor test. The results showed that the equation for the first single factor was 18.184%, which met the requirement of less than 50%.

### The Multiple Mediating Effects of Information Overload and Anxiety

**FIGURE 2:**
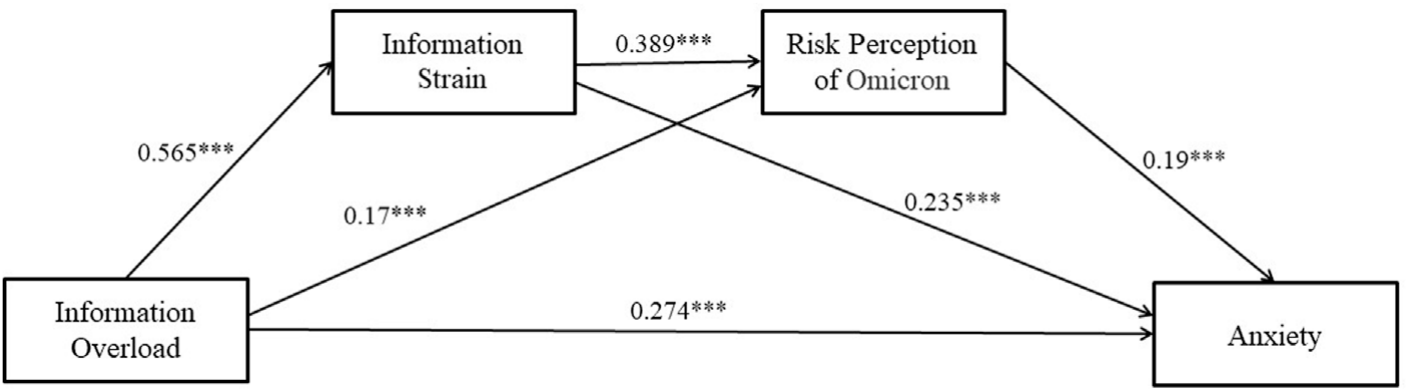
The chain mediating effect of information overload and anxiety (Shanghai, China. 2022). Note: ***p < .01.

Controlling the four types of control variables, mediating effects tests were conducted using model 6 by macro PROCESS4.0, and the results are presented in Figure 2. In step1, information overload significantly and positively affected information strain (*β* = .565, *p* = .000); in step2, information overload (*β* = .170, *p* = .000) and information strain (*β* = .389, *p* = .000) significantly and positively affected risk perception of Omicron; in step3, information overload (*β* = .274, *p* = .000), information strain (*β* = .235, *p* = .000), and risk perception of Omicron (*β* = .190, *p* = 000) each significantly and positively affected anxiety; in step4, information overload significantly and positively affects anxiety (*β* = .480, *p* = .000), which is significant at the 1% significance level, indicating that information overload has a significant positive effect on anxiety and supporting hypothesis H1a.

The test results of the mediation effect (see Figure 2) showed that information strain and risk perception of Omicron mediated the relationship between information overload and anxiety (supporting hypotheses H2a and H3a), and there was a chain mediation mechanism (hypothesis H4a is supported). Specifically, information overload affects anxiety in four ways: (a) Information Overload→ Anxiety; (b) Information Overload -> Information Strain -> Anxiety; (c) Information Overload -> Anxiety; (c) Information Overload -> Risk Perception of Omicron -> Anxiety; (d) Information Overload -> Information Strain -> Risk Perception of Omicron -> Anxiety. Thus, pathway (d) proves the existence of a chained mediation mechanism.

### Total Effect, Direct Effect, and Indirect Effect of the Chain Mediating Effect Between Information Overload and Anxiety

After the verification of the chain mediating effect played by information strain and risk perception of Omicron, we proceeded to calculate the total effect, direct effect, and indirect effect of the chain mediating effect (see Table 2). The results show that the total indirect effect (.207) accounts for 43.13% of the total effect (.480) and 75.55% of the direct effect (.274) in the relationship between information overload and anxiety. In other words, 43.13% of the effect of information overload exerting a positive influence on anxiety was acting through three mediating effects. Specifically, they are (a) the mediating effect of information strain, (b) the mediating effect of risk perception of Omicron, and (c) the chain mediating effect of information strain and risk perception of Omicron. Among them, the mediating effects (a), (b), and (c) reached 27.71%, 6.67%, and 8.75% of the total effect and 48.54%, 11.68%, and 15.33% of the direct effect, respectively. It can be seen that in the relationship between information overload and anxiety, the mediating effect of information strain is significantly stronger than that of risk perception of Omicron and the chain mediating effect. Moreover, the above tests of the total effect, direct effect and indirect effect, and mediating effects (a), (b), and (c) are all statistically significant at 95% confidence intervals that do not overlap with 0.

**TABLE 2:** Total effect, direct effect and indirect effect of the multiple mediating effect (Shanghai, China. 2022).

	Effect	Boot SE	Boot LLCI	Boot ULCI	Ratio of indirect to total effect	Ratio of indirect to direct effect
Total effect	.480	.036	.409	.552	–	–
Direct effect	.274	.040	.194	.353	–	–
Indirect effect	.207	.027	.154	.261	43.13%	75.55%
Ind1	.133	.027	.081	.187	27.71%	48.54%
Ind2	.032	.011	.013	.056	6.67%	11.68%
Ind3	.042	.011	.022	.065	8.75%	15.33%

Note: Ind1 is the mediation effect model of Information Overload -> Information Strain -> Anxiety, Ind2 is the mediation effect model of Information Overload -> Risk Perception of Omicron -> Anxiety, and Ind3 is the mediation effect model of Information Overload -> Information Strain -> Risk Perception of Omicron -> Anxiety. Boot SE, Boot LLCI and Boot ULCL is estimated standard error under bias-corrected percentile bootstrap method, and 95% confidence interval lower and 95% confidence interval upper, and Boot LLCI and Boot ULCL do not overlap with zero, number of bootstrap samples for percentile bootstrap confidence intervals is 5000.

### The Multiple Mediating Effects of Social Overload and Anxiety

**FIGURE 3:**
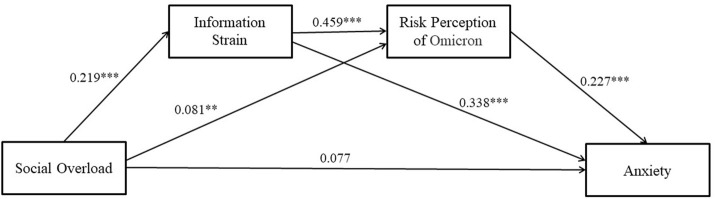
The chain mediating effect of social overload and anxiety (Shanghai, China. 2022). Note: ***p < .01, **p < .05.

In this section, we conducted a mediating effects test using model 6 of macro PROCESS4.0 after controlling the four types of control variables, and the results are presented in Figure 3. In step1, information overload significantly and positively affected information strain (*β* = .219, *p* = .000); in step2, social overload (*β* = .081, *p* = .042) and information strain (*β* = .459, *p* = .000) significantly and positively influenced risk perception of Omicron; in step3, information strain (*β* = .338, *p* = .000) and risk perception of Omicron (*β* = .227, *p* = .000) significantly and positively affected anxiety, but social overload did not significantly affect anxiety; in step4, social overload significantly and positively affected anxiety (*β* = .192, *p* = .000), indicating that social overload has a significant positive effect on anxiety, significant at 1% level of significance, and supporting hypothesis H1b.

The test results of the mediation effect (see Figure 3) showed that information strain and risk perception of Omicron mediated the relationship between social overload and anxiety (supporting hypotheses H2b and H3b), and there was a chain mediation mechanism (supporting hypothesis H4b). Specifically, there are four pathways at play in the process of social overload influencing anxiety: (a) Social Overload→ Anxiety; (b) Social Overload -> Information Strain -> Anxiety; (c) Social (d) Social Overload -> Risk Perception of Omicron -> Anxiety; (d) Social Overload -> Information Strain -> Risk Perception of Omicron -> Anxiety. Therefore, pathway (d) demonstrates the existence of a chain mediating mechanism.

### Total Effect, Direct Effect, and Indirect Effect of the Chain Mediating Effect Between Social Overload and Anxiety

After we verified the chain mediating effect played by information strain and risk perception of Omicron, we proceeded to calculate the total effect, direct effect, and indirect effect of the chain mediating effect (See Table 3). The results showed that the total indirect effect (.115) accounted for 59.90% of the total effect (.192) and 38.54% of the direct effect (.077) in the relationship between social overload and anxiety. That is to say, at a time when information overload exerts a positive effect on anxiety, 38.54% of the effect is mediated through three mediating effects. Specifically, they are (a) the mediating effect of information strain, (b) the mediating effect of risk perception of Omicron, and (c) the chain mediating effect of information strain and risk perception of Omicron. Among them, the mediating effects (a), (b), and (c) reached 38.54%, 9.38%, and 11.98% of the total effect, and 96.10%, 23.28%, and 29.87% of the direct effect, respectively. It can be seen that in the relationship between social overload and anxiety, the mediating effect of information strain is significantly stronger than that of risk perception of Omicron and chain mediation. In addition, the above tests of the total effect, direct effect and indirect effect, and mediating effects (a), (b), and (c) are all statistically significant at 95% confidence intervals that do not overlap with 0.

**TABLE 3:** Total effect, direct effect and indirect effect of the multiple mediating effect (Shanghai, China. 2022).

	Effect	Boot SE	Boot LLCI	Boot ULCI	Ratio of indirect to total effect	Ratio of indirect to direct effect
Total effect	.192	.045	.103	.281	–	–
Direct effect	.077	.040	−.002	.156	–	–
Indirect effect	.115	.025	.067	.164	59.90%	149.35%
Ind1	.074	.020	.037	.115	38.54%	96.10%
Ind2	.018	.010	.000	.041	9.38%	23.38%
Ind3	.023	.007	.011	.037	11.98%	29.87%

Note: Ind1 is the mediation effect model of Social Overload -> Information Strain -> Anxiety, Ind2 is the mediation effect model of Social Overload -> Risk Perception of Omicron -> Anxiety, and Ind3 is the mediation effect model of Social Overload -> Information Strain -> Risk Perception of Omicron -> Anxiety. Boot SE, Boot LLCI and Boot ULCL is estimated standard error under bias-corrected percentile bootstrap method, and 95% confidence interval lower and 95% confidence interval upper, and Boot LLCI and Boot ULCL do not overlap with zero, number of bootstrap samples for percentile bootstrap confidence intervals is 5000.

## Discussion

Using an online survey, the present study explored the factors and mechanisms of anxiety of college students during the Omicron wave lockdown in Shanghai, China. Specifically, the current study validated the underlying mechanism of the relationship between social media overload and anxiety among university students. In line with our assumption, both social overload and information overload were significantly and positively related to anxiety. One study found that pandemic experience may affect internalizing symptoms in university students during the early months of the pandemic [61]. Our findings showed that too much information and socializing on social media will increase people’s anxiety. Although users can get social support from social media, social requests that exceed one’s ability to handle will also have negative consequences. When public health emergencies become social themes among social media users, they will have a negative impact on mental health. This finding is different from Marzouki’s research that social media use could significantly reduce individuals’ anxiety during the first 9 weeks of worldwide lockdown[62]. In SNS usage, the threshold point of information amount is regarded as the transition point from a non-overload state to an overload state[28]. This study found that when the amount of information and social support reaches the threshold point and leads to overload, the buffer effect of social media use on anxiety will disappear, or even make an opposite effect. Even though the position of the threshold point needs further exploration, this result gives new sight of how social media use make different effects on mental health.

To the best of our knowledge, this is the first study that examined the mediating role of information strain in the relationship between social media overload and anxiety. Online social networks have been regarded as a source and symbol of stress [63]. Prior research has proved that technostress may induce emotional reactions [64]. Yang and Lin [19] found that the use of information and communication technology may cause technostress to users, resulting in a state of anxiety or tension [19]. In this paper, when a person feels more socially overloaded, there is greater information stress, which may lead to higher levels of anxiety. A possible explanation may be that information beyond the filtering and processing capacity will cause pressure on college students. In addition, too much social support and negative information content may also predict stress related to information.

In line with our assumption, the risk perception of Omicron mediated the association between social media overload and anxiety. The finding is consistent with previous studies that perceived COVID-19 information overload influenced Gen Z’s fear of COVID-19 [65]. Lots of empirical evidence showed that risk perception was regarded as a predictor of anxiety in different kinds of pandemic illnesses, such as SARS [66], H1N1 [67], Ebola virus [68]. This finding provides new empirical evidence for a social amplification theory of risk. Specifically, social media overload amplifies risk perception in risk communication. A possible explanation may be in two ways, on the one hand, information seeking after the COVID-19 pandemic helps to reduce differences and promote consensus [62]. Users with similar interests on social media come together and eventually form a homogeneous group, known as “echo chamber” [69]. On the other hand, the use of algorithmic on social media increase the information cocoon room. In the event of public health emergencies, the information cocoon room strengthened the consensus on risks by pushing homogeneous content and triggered negative emotions such as anxiety [70]. Therefore, Homogenized COVID-19 risk information increases, resulting in high levels of risk perception in individuals, which causes negative effects on mental health.

Further, the chain mediating role of information strain and risk perception of Omicron in the relationship between social overload and anxiety has been proved. This finding suggests that when people experience stronger social media overload, they will feel increased information strain and perceive a higher risk of Omicron, and these feelings increase the level of anxiety. Higher emotional stress predicted higher levels of risk perception [71]. Strain may lead to negative emotions such as anger and frustration [72]. A study pointed out that perceived risk is an important predictor of stress in a catastrophe risk situation [73]. This finding provides new evidence that strain may also lead to increased risk perception.

The current manuscript explores the internal mechanism of the impact of social media overload on anxiety. However, the present study still has some limitations. First, the data in this paper were collected at the beginning of the quarantine period, there was a lack of data at the peak and later stages of the pandemic. Future research could explore the impact of social media use on mental health at different stages of public health events. Second, this is a cross-sectional study, our empirical analysis can only reflect correlations and cannot deduce causality. Third, the current manuscript is a study of college students in the particular context of the school lockdown in Shanghai, the research on the relationship between social overload and anxiety could be extended to a wider group in the future. In fact, the use of social media by other vulnerable groups, such as the elderly who face the digital gap, is worth paying attention to as well as mental health issues. In public health events, whether the lack of social media digital skills will affect mental health is also worth further discussion. And finally, due to the constraints in the data collection process, the order of the measured variables was not taken into account, and this may have had an impact on our findings.

Despite these limitations, our study has several strengths. First, most of the current literature focuses on mental health at the beginning of the COVID-19 pandemic, our investigation reflects the most up-to-date situation of college students’ mental health during the latter part of the COVID-19 pandemic. Second, this study adds to the knowledge of the literature on how digital information media, represented by social media, played a role in psychological anxiety and provides new insights for further assessment and treatment practices for digital mental health interventions. Information overload and social overload are predictors of mental health problems. Although information access and social support are very important during the pandemic, excessive information and social interaction should be avoided to reduce information strain and risk perception and promote public mental health. Finally, this study also provides new supporting evidence for the social amplification theory of risk, where a large volume of social media information influenced respondents’ risk perceptions by a short period of time, and this risk perception then led to negative psychological anxiety.

### Conclusion

Under China’s zero-Covid strategy, college students in Shanghai experienced campus lockdown. During this period, the mental health of college students has aroused concern. This study provides valuable insights into the impact of social media use on people’s mental health during the pandemic. The present study found that social media overload (including information overload and social overload) is a risk predictor of anxiety. Information strain and risk perception of Omicron mediated this association. Besides, this manuscript also conducts a chain mediating role of information strain and risk perception in the relationship between social media overload and anxiety. The results would suggest that people need to pay attention to social media overload at public health events. The media should release information in time to clear up the rumors. This results also suggest appropriate use of social media may avoid the resulting negative effects on mental health. Users should take some self-intervention measures, such as controlling the time and frequency of social media use, in order to avoid social media overload. Furthermore, governments and relevant institutions should provide appropriate psychological services for individuals.
